# Guidelines for the Bandgap Combinations and Absorption Windows for Organic Tandem and Triple-Junction Solar Cells

**DOI:** 10.3390/ma5101933

**Published:** 2012-10-22

**Authors:** Ben Minnaert, Peter Veelaert

**Affiliations:** Faculty of Applied Engineering Sciences, University College Ghent—Ghent University, Valentin Vaerwyckweg 1, Gent B-9000, Belgium; E-Mail: Peter.Veelaert@UGent.be

**Keywords:** organic solar cells, modeling, tandem solar cells, triple-junction solar cells, multi-junction solar cells, power conversion efficiency, bandgap, absorption window

## Abstract

Organic solar cells have narrow absorption windows, compared to the absorption band of inorganic semiconductors. A possible way to capture a wider band of the solar spectrum—and thus increasing the power conversion efficiency—is using more solar cells with different bandgaps in a row, *i.e.*, a multi-junction solar cell. We calculate the ideal material characteristics (bandgap combinations and absorption windows) for an organic tandem and triple-junction solar cell, as well as their acceptable range. In this way, we give guidelines to organic material designers.

## 1. Introduction

Photovoltaic (PV) solar cells based on organic compounds are promising candidates for solar energy conversion. They have the potential for cost effectiveness, mechanical flexibility and easy processing. Nowadays, a record efficiency of 10% is reached [1] for a single-junction cell of 1 cm^2^ and 4.2% for a submodule (10 series cells) [2,3]. In order to compete with the traditional inorganic cells, higher power conversion efficiencies, certainly for larger cells, are desirable.

A characteristic of organic solar cells is their narrow absorption window, compared to the absorption band of inorganic semiconductors [4]. A possible way to capture a wider band of the solar spectrum—and thus increasing the power conversion efficiency—is using more solar cells with different bandgaps in a row, referred to as a tandem or multi-junction solar cell. In this article, we will focus on organic multi-junction solar cells with two or three cells in a row. We will refer to them as a tandem (2 subcells) and a triple-junction (3 subcells) solar cell.

Organic tandem solar cells, where both single subcells are of the organic solar cell type, have already been fabricated by several research institutes [5,6,7,8,9,10,11,12] as well as fully organic triple-junction cells [13,14,15]. Nowadays, efficiencies of more than 10% are reached for organic tandem cells [16,17]. This maximum efficiency is about the same as the record efficiency of a single-junction organic cell, indicating that progress in the field of multi-junction organic solar cells is still possible.

In this paper, we calculate the ideal material characteristics for an organic tandem and triple-junction solar cell. In this way, we give guidelines to organic material designers and tell them what can be the expected result of their new developed material if it would be used in an organic multi-junction solar cell. More specifically:

- We determine the optimal bandgap configurations of the subcells, as well as their acceptable range.
- Previous excellent work on calculating the ideal configuration of organic (single and multi-junction) solar cells has been done by multiple authors [18,19,20,21,22,23,24,25,26], but often, they do not take into account the narrow absorption window, characteristic for organic materials. In this work, we include the influence of different absorption windows for each subcell.
- Moreover, our calculations with different absorption windows are not only presented for a stacked organic tandem and triple-junction cell, but also for a monolithic configuration. This has not been done before for organic triple-junction cells.
- We calculate the theoretical upper-limit for the efficiency of organic tandem and triple-junction solar cells. Although this maximum efficiency itself is only interesting from a theoretical point of view, the ideal material characteristics obtained from these calculations are of importance. They tell material designers what the sufficient energy levels and absorption windows for organic photovoltaics are.
- We also assume a more realistic scenario to predict efficiencies obtainable in the near future.

The results presented in this paper are meant to increase the fundamental understanding of the relationship between on the one hand the energy levels of donor and acceptor and the absorption window of the subcells, and on the other hand the light harvesting potential of the configurations. This work is an extension of our earlier work on organic multi-junction cells [27,28].

## 2. Improving the Efficiency of Organic Solar Cells

The active material in a single organic bulk heterojunction solar cell consists of an interpenetrating network of an *n*-type (electron acceptor, e.g., fullerene derivative) and a *p*-type (semi)conductor (electron donor, e.g., conjugated polymer), sandwiched between two electrodes with different work functions. The optical bandgap *E_g_* is defined as the difference between the lowest unoccupied molecular orbital (LUMO) and the highest occupied molecular orbital (HOMO) of the absorber material.

**Figure 1:**
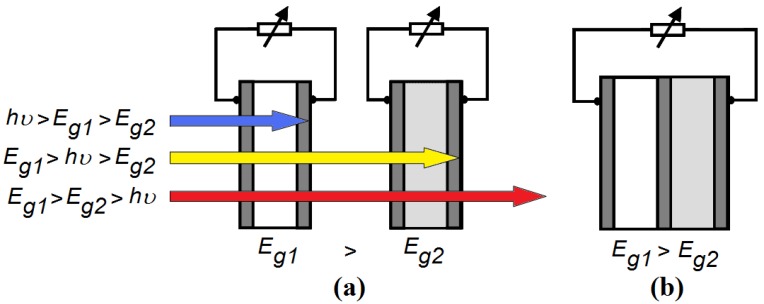
(**a**) A stacked or 4-terminal tandem solar cell: the first single cell absorbs photons with an energy *hν* higher than *E_g1_*. The second cell absorbs photons with an energy between *E_g1_* and *E_g2_*. Photons with an energy below *E_g2_* are not absorbed. The two subcells are electrically separated; (**b**) A monolithic or 2-terminal tandem solar cell: the single cells are electrically connected in series.

The power conversion efficiency of a solar cell can be significantly increased by using several solar cells with different bandgaps *E_g_* in a row. We consider a tandem solar cell, consisting of two single organic photovoltaic cells (Figure 1a). The organic cell with the highest optical bandgap is in front (side of the sun), thus *E_g1_*> *E_g2_*. High-energy photons with an energy *hν*>*E_g1_* are absorbed by the first cell. The second cell, with a lower bandgap *E_g2_*, absorbs the low-energy photons with an energy between *E_g1_* and *E_g2_*. In this configuration, the photon energy is used more efficiently: the voltage at which electrical charge is collected in each subcell is closer to the energy of the photons absorbed in that subcell.

In the ideal configuration, the subcells are electrically separated. This is called the stacked or 4-terminal configuration (Figure 1a). However, the stacked configuration is to date economically irrelevant. Indeed, experimental and commercial tandem solar cells are usually of the monolithic (integrated or 2-terminal) type (Figure 1b). This means that they are not only optically in series, but also electrically in series. This configuration will never reach an efficiency that is higher than that of a stacked (4-terminal) tandem cell, because all single cells cannot operate at their optimal working point at the same time (unless they have an equal maximum-power current).

Chemists are searching for suitable organic materials, and with appropriate actions (the *tuning* or *molecular engineering* of organic molecules), they improve the current materials for photovoltaic applications: And with success. The power conversion efficiency of organic single-junctions cells has improved from 1% in 1985 [29], to 5% in 2005 [30], and to 10% nowadays [1]. Appropriate actions to modify the properties of organic materials are, for example:

- The substitution of, or addition on, side groups of the organic chain.
- Changing the chain lengths between the atoms (e.g., by adding double bonds).
- Changing the aromaticity of the bounds.
- Modifying the spatial orientation (planarity).

An example of *tuning* or *molecular engineering* of organic molecules is the insertion of extra double bounds in an oligothiophene structure, thus lowering the bandgap [31]. Not only the active material, but also the solar device itself is the subject of *molecular engineering*. A clear example can be found in [32] where electron-acceptor side groups are added in different positions on a molecule. A clear relationship can be found between, on the one hand, the type and location of the side group, and, on the other hand, the open circuit voltage and the quantum efficiency *QE* (both in terms of shape of the *QE*-graph, and of the width of the absorption window).

For organic single-junction cells, it has already been demonstrated that lower bandgaps and broader absorption windows are necessary to improve the efficiency [33,34]. In the next sections, we study to what extend the same is true for multi-junctions. We determine where the focus should be laid. For example, can a larger efficiency gain be achieved in the first place by broadening the absorption window, or by adjusting the energy levels?

## 3. Assumptions of the Model

**Figure 2:**
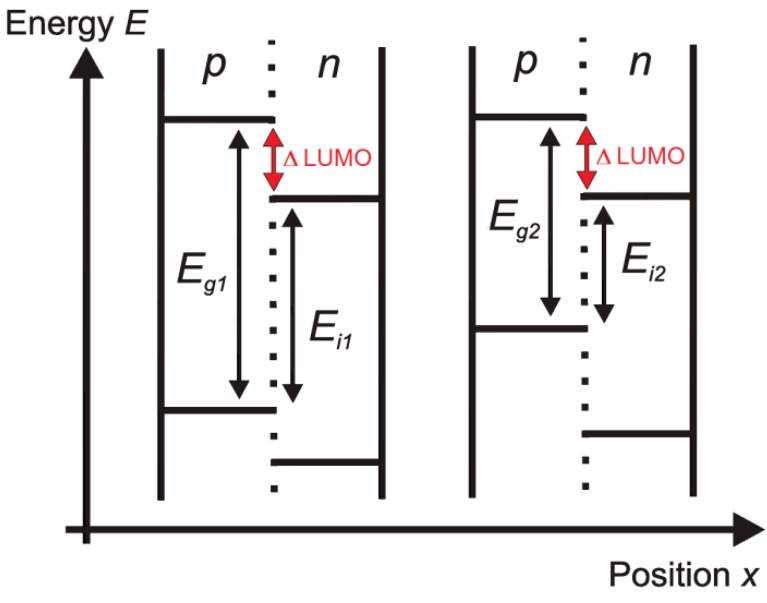
The schematic energy band diagram of a stacked organic tandem solar cell. Only the *p*-material (donor) is the absorber. The mutual position of the single cells does not matter, because the cells are only optically and not electrically connected in series. The absorber bandgap *E_g_*, the interface bandgap *E_i_*, and the difference between the LUMO-levels of each subcell are indicated.

We consider a 4-terminal tandem solar cell, consisting of two single organic photovoltaic cells (see Figure 2 for the schematic energy band diagram). We assume that in each single cell, only one material absorbs light (which is a good approximation [35]). Usually, most of the light is absorbed by the *p*-type component (donor); this is the case we will consider here onwards. In the other case, when the *n*-type material (acceptor) absorbs all light, the results remain the same by permutation of *n* and *p* [34]. When both materials absorb light, the highest maximum attainable efficiency reached is the same as in the case where only one material absorbs light, but higher efficiencies are reached for materials which have not optimal energy levels [34].

Because we want to investigate the relationship between on the one hand the energy levels of donor and acceptor and the absorption window of the subcells, and on the other hand the light harvesting potential of the configurations, we assume full absorption in each subcell (and consequently leaving the thickness of the subcells aside). We neglect interference and optical coupling of the subcells, thus overestimating the efficiency potential. The organic cell with the widest absorber bandgap is at top (at the side of the sun), thus *E_g1_* > *E_g2_*. The distance between the HOMO of the *p*-type and the LUMO of the *n*-type is considered as the thermodynamic limitation of the useful energy [36]. We call this value the interface bandgap *E_i_*. For an organic solar cell with ohmic contacts, the open circuit voltage *V_oc_* is linearly dependent on the interface bandgap *E_i_*. This linear relationship was proven for the variation of the HOMO-level of the donor [18,37,38] and of the LUMO-level of the acceptor [39,40,41]. For a cell with non-ohmic contacts, the *V_oc_* is dependent on the work function difference of the electrodes [42]. In our calculations, we assume a cell with ohmic contacts.

For our simulations, the following fundamental assumptions are made about the stacked tandem cell (Figure 1a):

- Every photon with an energy *h*ν higher than the bandgap *E_g1_* is absorbed by the first cell and leads to a useful energy *E_i1_*. This assumption implies that each absorbed photon eventually leads to a free electron and a free hole, with an energy difference of *E_i1_* between them.
- Every photon with an energy *h*ν between *E_g1_* and *E_g2_* is absorbed by the second cell and leads to a useful energy *E_i2_*.
- Photons with an energy *h*ν lower than *E_g2_* are fully transmitted.

**Figure 3:**
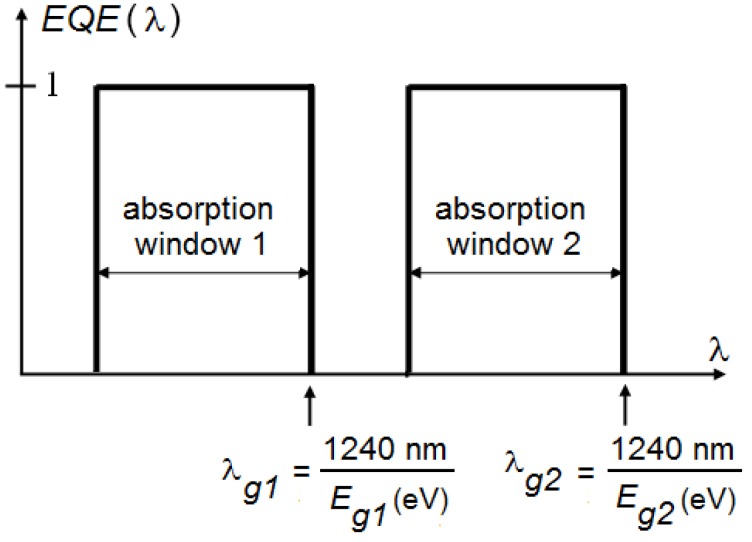
External quantum efficiency (*EQE*) as a function of the wavelength λ. Definition of the absorption window and the cut-off wavelengths λ_g_. Notice that the order of the first and second subcell can be changed if there is no overlap between both absorption windows.

The maximum efficiency *η_max_* is therefore given by:

(1) $$\eta_{\max}=\frac{E_{i1}\int_{E_{g1}}^{\infty }N(E)\;dE+E_{i2}\int_{E_{g2}}^{E_{g1}}N(E)\;dE}{\int_{0}^{\infty }E\;N(E)\;dE},\;with\;\;E_{g1}>E_{g2}$$

with *N*(*E*) the incident photon flux. For all our simulations, we use the AM 1.5 experimentally measured solar spectrum [43]. Note that the denominator is the total incident photon power density of the solar spectrum and does not depend on any bandgap. In this ideal scenario, the open circuit voltage *V_oc_* of the first and second subcell will be given by *E_i1_/q* and *E_i2_/q* respectively (with *q* the electric charge). The fill factor *FF* of both subcells is assumed to equal unity, as well as the external quantum efficiency *EQE* of the first cell for wavelengths below the cut-off wavelength λ_g1_ (corresponding with *E_g1_*, see Figure 3). The *EQE* of the second cell equals unity for wavelengths between cut-off wavelength λ_g1_ and λ_g2_ (corresponding with *E_g2_*). Because these parameters are scalable, this idealization does not interfere with our goal to determine an acceptable range for the bandgap combinations and absorption windows.

For now, our assumptions correspond with a typical inorganic cell where all energies above the bandgap are absorbed. We will now refine our model for organic solar cells by imposing a narrow absorption band as well as a difference between the LUMOs of donor and acceptor. In real organic materials, the optical absorption and hence the *EQE* are confined to a more or less narrow wavelength range, usually about 200 to 300 nm wide. We idealize this behavior by introducing the concept of absorption windows [34], which are defined in Figure 3, and will be treated further as a parameter.

In a monolithic or integrated tandem solar cell (Figure 1b), the individual cells are electrically connected in series. This means that the total voltage over the cell is the sum of the voltages over each individual cell, and thus equals the sum of the interface bandgaps of both single cells. Furthermore, the same current flows through both single cells. Hence, the maximum efficiency *η_max_* for a monolithic organic tandem cell is given by:

(2) $$\eta_{\max}=\frac{\left(E_{i1}+E_{i2})\cdot \min(\int_{E_{g1}}^{\infty }N(E)\;dE,\int_{E_{g2}}^{E_{g1}}N(E)\;dE\right)}{\int_{0}^{\infty }E\;N(E)\;dE},\;with\;\;E_{g1}>E_{g2}$$

with min(*x*,*y*) the minimum of *x* and *y*. The open circuit voltage *V_oc_* of the whole monolithic tandem cell will be given by (*E_i1_+E_i2_)/q*, the fill factor *FF* equals unity, as does the external quantum efficiency *EQE* for wavelengths below the cut-off wavelength λ_g2_.

In organic bulk heterojunction solar cells, light absorption does not immediately lead to free charge carriers. Instead, an exciton is created. In an ideal scenario, the highest efficiency is reached when the LUMO of the *p*-material is as close as possible to the LUMO of the *n*-material [34]. However, a necessary condition for efficient dissociation of the created excitons is that the difference between the LUMOs of donor and acceptor (ΔLUMO, Figure 2) is higher than the exciton binding energy [44]. Thus, without a sufficient energy difference between the LUMOs of both materials, the solar cell cannot work. The value of the exciton binding energy (and the minimal ΔLUMO) in different materials is a subject of discussion, and values in a large range from 0.1 eV to 2 eV have been published [39,41,45,46]. The excess of this necessary minimum of the LUMO-difference corresponds with an energy loss. We refer to our earlier work [34] for the influence of ΔLUMO on the efficiency for different absorption windows for a single-junction. With a full absorption window, each additional difference of 0.1 eV between the LUMOs results in approximately an additional 10% relative efficiency loss. In the following calculations, we assume a difference of 0.2 eV between the LUMOs of each subcell in our organic solar cell. This value was put forward as an empirical threshold necessary for exciton dissociation [47] and is comparable with other studies [18,24]. Just because of this necessary energy difference between the LUMOs, the attainable efficiency for the organic bulk heterojunction tandem solar cell drops by 16%–17% in comparison with their inorganic counterpart, purely because of the difficulties in exciton dissociation. Choosing another value for ΔLUMO would lead to qualitative similar results.

We also consider an organic triple-junction solar cell, *i.e.*, three organic subcells in a row. We make completely analogous assumptions as for the tandem cell. Formulae (1) and (2) now become

(3) $$\eta_{\max}=\frac{E_{i1}\int_{E_{g1}}^{\infty }N(E)\;dE+E_{i2}\int_{E_{g2}}^{E_{g1}}N(E)\;dE+E_{i3}\int_{E_{g3}}^{E_{g2}}N(E)\;dE}{\int_{0}^{\infty }E\;N(E)\;dE},\;with\;\;E_{g1}>E_{g2}>E_{g3}$$

for a stacked and

(4) $$\eta_{\max}=\frac{\left(E_{i1}+E_{i2}+E_{i3})\cdot \min(\int_{E_{g1}}^{\infty }N(E)\;dE,\int_{E_{g2}}^{E_{g1}}N(E)\;dE,\int_{E_{g3}}^{E_{g2}}N(E)\;dE\right)}{\int_{0}^{\infty }E\;N(E)\;dE},\;with\;\;E_{g1}>E_{g2}>E_{g3}$$

for a monolithic triple-junction configuration, respectively.

In the next section, we first take a look at the special case where all the subcells of the solar cell have a maximum absorption window. Secondly, we discuss the case where the subcells have the same, narrow absorption window. Thirdly, an organic cell with different absorption windows for the subcells is considered. Finally, a more realistic scenario is discussed.

## 4. Results

### 4.1. Subcells with a Full Absorption Window

**Figure 4:**
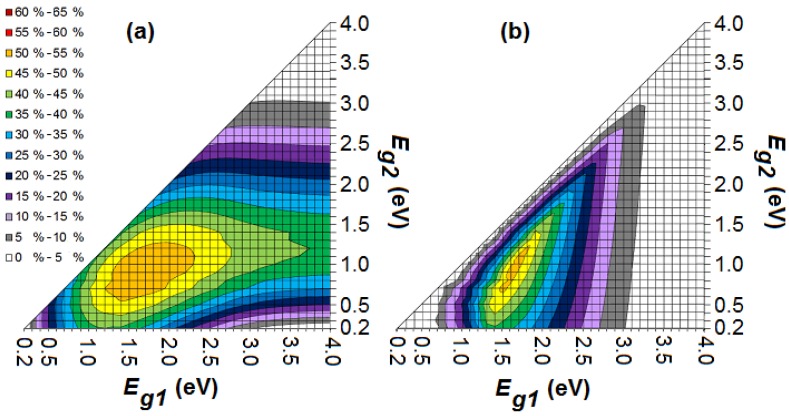
The maximum efficiency in the ideal scenario for a stacked (**a**) and monolithic (**b**) organic tandem cell with bandgaps *E_g1_* and *E_g2_*, a full absorption window and a LUMO difference of 0.2 eV for each subcell between *n*- and *p*-type.

Figure 4 shows the maximum efficiency in the ideal scenario for a stacked and monolithic organic tandem cell with bandgaps *E_g1_* and *E_g2_*, a full absorption window for the subcells and a LUMO difference of 0.2 eV for each subcell between *n*- and *p*-type. A maximum efficiency of 54.0% and 53.3% is reached for a stacked and monolithic tandem cell respectively. As mentioned already, the efficiency of a monolithic configuration will never be higher than that of a stacked configuration. In comparison with a single-junction organic cell with an optimal bandgap of 1.1 eV, adding a second subcell results in a relative gain of about 1/3rd in power conversion efficiency [34]. For higher bandgaps, less photons are being absorbed from the solar spectrum, but the useful output energy of each absorbed photon is higher. Hence, there is an optimum for each bandgap. This maximum occurs for the stacked and monolithic tandem cell at a configuration (*E_g1_*, *E_g2_*) of (1.7 eV, 0.9 eV) and (1.6 eV, 0.9 eV) respectively [27]. Several organic donors with a bandgap of 1.6 or 1.7 eV are available (e.g., CuPc, P3OT and PBDTTT-CF) [24,47]. However, although organic materials with a bandgap of 0.9 eV exist [33], they have not (yet) been applied successfully in an organic solar cell.

**Figure 5:**
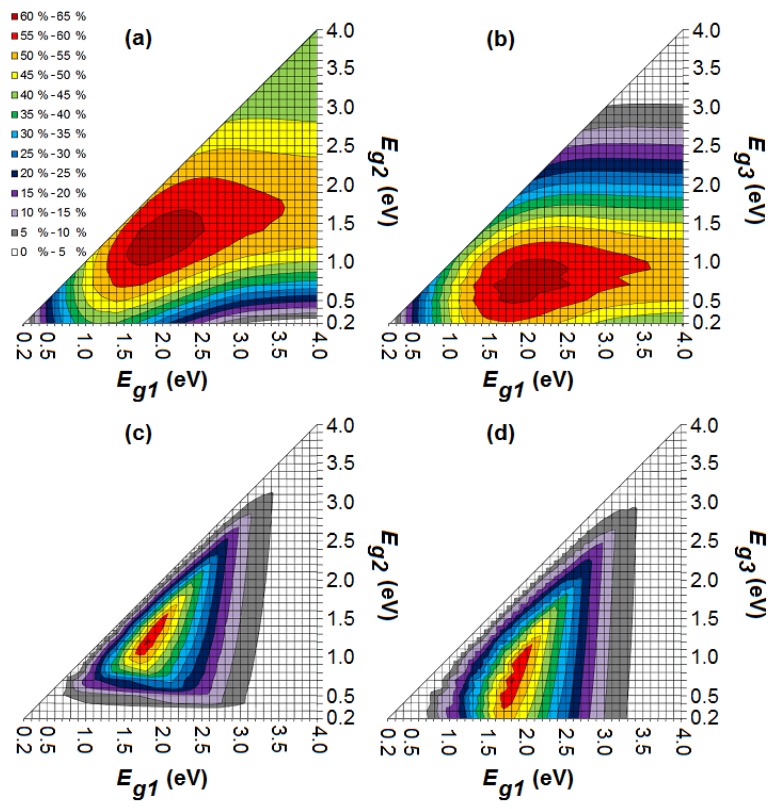
The maximum efficiency in the ideal scenario for a stacked (**a**,**b**) and monolithic (**c**,**d**) organic triple-junction solar cell with bandgaps *E_g1_*, *E_g2_* and *E_g3_*, a full absorption window and a LUMO difference of 0.2 eV between *n*- and *p*-type.

For the triple-junction solar cell, a maximum efficiency of 62.2% and 61.0% is reached for a stacked and monolithic cell respectively (Figure 5). In comparison with a single-junction organic cell, adding two subcells results in a relative gain of more than 50 % in power conversion efficiency. If we compare the triple-junction with the tandem solar cell, adding a third subcell results in a relative gain of about 15% in power conversion efficiency. Figure 5 reveals the optimum (*E_g1_*, *E_g2_*, *E_g3_*) configuration for the triple-junction: (1.9 eV, 1.2 eV, 0.7 eV) and (1.8 eV, 1.2 eV, 0.7 eV) for the stacked and monolithic cell respectively. One of the most successful donors for organic PV, *i.e.*, P3HT, has a bandgap of 1.9 eV. Also organic materials with a bandgap of 1.8 eV (e.g., PCDTBT) and 1.2 eV (e.g., P3TPQ and PTBEHT) are suitable for organic PV [24,33]. The problem is again the last subcell. Although a conjugated polymer with a bandgap of 0.7 eV exists [48], it has not yet been applied successfully in a solar cell.

The requirements for a close to optimal configuration of the stacked cell are for *E_g1_* and *E_g2_* quite broad, permitting that the values of those bandgaps for optimal cells are not that strict. However, for *E_g3_*, this is not the case. A low bandgap *E_g3_* is necessary for a good device. For a monolithic configuration, the requirements are much stricter: all three bandgaps have only a small region wherein the power conversion efficiency of the solar cell is high.

Most organic absorbers have a wide bandgap and the production of suitable organic absorbers for photovoltaic applications with a low bandgap is problematic [4]. If we consider an organic tandem cell with optical bandgaps *E_g1_* = 2.5 eV and *E_g2_* = 1.5 eV, the stacked cell still has a maximum efficiency of 43.6%, whereas the monolithic cell only reaches 22.4%. We may conclude that a monolithic tandem cell is much less efficient than a stacked cell in a non-optimal bandgap configuration. For an optimal bandgap configuration, however, the difference is negligible. The same applies to the triple-junction. This means that for the production of tandem and triple-junction cells, the choice of good bandgap combinations (and thus material combinations) is much more limiting for a monolithic configuration than it is for a stacked configuration.

The current extracted from the monolithic configuration is almost equal to the photocurrent of the subcell that generates the lowest current. If one subcell generates much more current than another subcell, the excess of charge carriers cannot recombine at the intermediate contact between the subcells. This will cause a charging at the intermediate contact and will partially compensate the built-in voltage across the other cell until the current of the subcells matches. This will lower the power conversion efficiency and explains the inferior performance of monolithic cells for non-optimal bandgap configurations. Current matching is therefore necessary in a monolithic configuration. We want to stress that this is not implemented in the model presented in this paper.

### 4.2. Subcells with the Same Absorption Window 

We now take into account the narrow absorption window which is characteristic for organic materials. For ease of presentation, we assume—for now—that all subcells of the multi-junction structure have the same absorption window in nm.

**Figure 6:**
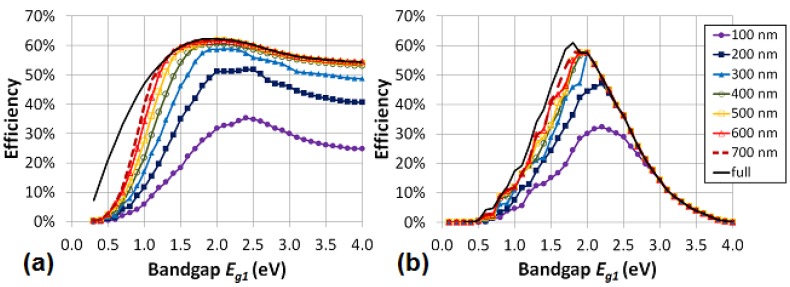
The maximum efficiency in the ideal scenario for a stacked (**a**) and a monolithic (**b**) organic triple-junction solar cell with varying bandgap *E_g1_*, optimal chosen bandgaps *E_g2_* and *E_g3_*, and a LUMO difference of 0.2 eV between *n*- and *p*-type. The absorption window varies from full absorption width to 100 nm absorption width.

First, as example, we consider the maximum efficiency for a stacked organic triple-junction solar cell with varying bandgap *E_g1_*, optimal chosen bandgaps *E_g2_* and *E_g3_* and a LUMO difference of 0.2 eV between *n*- and *p*-type. We vary the absorption window from full absorption width to 100 nm absorption width (Figure 6a). We notice two important results. First, the efficiency remains quite high, until the absorption width decreases under 300 nm. Secondly, the smaller the absorption window, the higher the optimal bandgap *E_g1_* of the first cell. The same two conclusions can be drawn for the monolithic configuration (Figure 6b). Notice again that the monolithic cell is much less efficient than a stacked cell in a non-optimal bandgap configuration. For the optimal bandgap configuration, the difference is again negligible.

**Figure 7:**
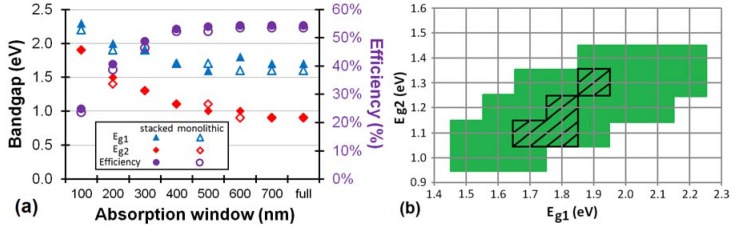
(**a**) The maximum efficiency is plotted in the ideal scenario (with ΔLUMO = 0.2 eV) for an organic stacked and monolithic tandem cell as a function of the absorption window. Also the optimum bandgaps *E_g1_* and *E_g2_* for a stacked cell are plotted as a function of the absorption window. The optimum bandgaps *E_g1_* and *E_g2_* for the monolithic cell are plotted if they differ from the stacked cell; (**b**) The green region and the black hatched region define the range of sufficient bandgap combinations (>90% of the maximum efficiency) of respectively a stacked and a monolithic organic tandem cell with absorption windows of 400 nm for the subcells.

**Figure 8:**
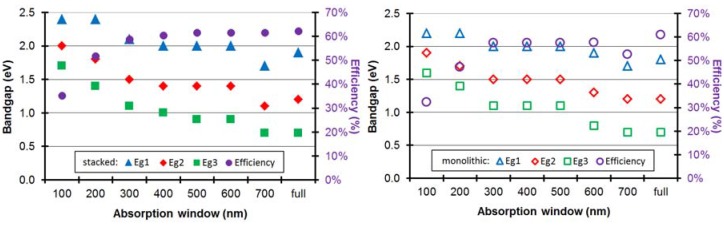
The maximum efficiency and the optimum bandgaps are plotted in the ideal scenario (with ΔLUMO = 0.2 eV) for an organic stacked and monolithic triple-junction cell as a function of the absorption window.

A more general view can be found in Figure 7a and Figure 8 which show the maximum efficiency and the optimum bandgaps in the ideal scenario for a stacked and monolithic configuration as a function of the absorption window width.

Let us first focus on the tandem cell (Figure 7a). The broader the absorption window, the higher the efficiency. Notice that there is only a negligible difference between the stacked and the monolithic configuration. As explained above, the efficiency for non-optimal bandgap configurations of the monolithic tandem cell will be much lower than for the stacked cell. For example, if we look at an organic tandem cell with non-optimal bandgaps *E_g1_* = 2.5 eV and *E_g2_* = 1.5 eV and an absorption window of 100, 200 and 300 nm, the stacked cell still has a maximum efficiency of 20.2, 32.5 and 41.5%, respectively, whereas the monolithic cell reaches only 18.0% for a 100 nm broad absorption window and 22.5% for an absorption window of 200 nm or more wide. Thus in the case of non-optimal bandgaps, we can conclude that for increasingly smaller absorption windows, the advantage of the stacked solar cell over the monolithic cell decreases.

Figure 7a shows that the optimum bandgap of the cells shifts towards higher energies for lower absorption windows. For example, the optimum bandgap shifts from *E_g1_* = 1.6 eV and *E_g2_* = 0.9 eV for a full absorption band monolithic cell to *E_g1_* = 1.9 eV and *E_g2_* = 1.4 eV for a cell with an absorption window of only 200 nm. This is a satisfying result, because, as we already mentioned, the production of suitable low bandgap organic materials is difficult.

For an absorption window of 400 nm or more, already 98% of the maximum attainable efficiency (for a full absorption band) is reached for the stacked and monolithic configuration. Hence, it would not pay off to try to develop organic materials with an absorption window broader than 400 nm, because hardly any efficiency gain can be achieved by widening the absorption window further. An absorption window of 400 nm is sufficient for an organic tandem cell.

The optimum bandgaps with a sufficient absorption window of 400 nm are *E_g1_* = 1.7 eV and *E_g2_* = 1.1 eV for both configurations. Fortunately, the maximum is quite broad, certainly for the stacked configuration, just as it was for Figure 4 for the full absorption band. If we consider a bandgap *E_g2_* of 1.4 eV (*E_g1_* = 2.2 eV) we still obtain 90% relative of the maximum efficiency for the stacked cell.

Figure 7b defines the range of sufficient energy levels for a good organic tandem solar cell. As mentioned earlier, a minimum absorption window of 400 nm is advised. We define this range as any bandgap combination which still achieves an efficiency of minimum 90% relative of the maximum efficiency at 400 nm. Figure 7b gives guidelines for the sufficient bandgap combinations for an organic tandem solar cell.

For the triple-junction cell (Figure 8), similar conclusions can be drawn. The broader the absorption window, the higher the efficiency. Only at an absorption window of 700 nm, the efficiency decreases because—by imposing the absorption window—we also impose limits on the allowed bandgaps. Indeed, a bandgap of e.g., 2.0 eV corresponds with a cut-off wavelength of 620 nm and does therefore not leave enough room for an absorption window of 700 nm.

The optimum bandgaps (*E_g1_*, *E_g2_*, *E_g3_*) for a stacked cell shift from (1.9 eV, 1.2 eV, 0.7 eV) for a full absorption band to (2.4 eV, 1.8 eV, 1.4 eV) for a cell with an absorption window of only 200 nm. For an absorption window of 300 nm respectively, already 95% of the maximum attainable efficiency (for a full absorption band) is reached. Hence, it would not pay off to try to develop organic materials for triple-junction solar cells with an absorption window broader than 300 nm, because hardly any efficiency gain can be achieved by widening the absorption window further. The optimum bandgaps with a sufficient absorption window of 300 nm are (2.1 eV, 1.5 eV, 1.1 eV) for the stacked and (2.0 eV, 1.5 eV, 1.1 eV) for the monolithic configuration.

**Figure 9:**
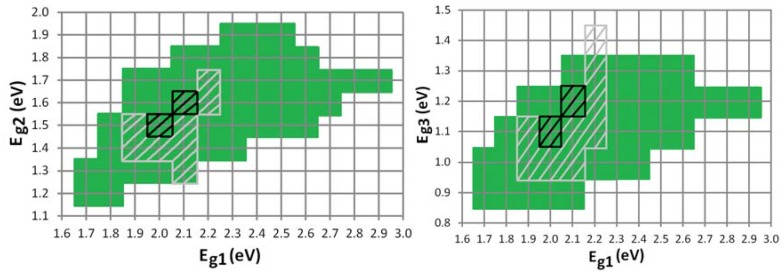
The green region and the black hatched region define the range of sufficient bandgap combinations (>90% of the maximum) of respectively a stacked and a monolithic organic triple-junction cell with absorption windows of 300 nm for the subcells. The grey hatched region defines the “still acceptable” range (>80% of the maximum) for a monolithic triple-junction.

Especially the optimum bandgap *E_g3_* of the third subcell is quite low for organic materials. If we again consider a range of sufficient bandgap combinations (Figure 9), we notice that for a stacked configuration, an *E_g3_* of 1.3 eV is still very acceptable for different *E_g1_*–*E_g2_* combinations. For a monolithic configuration however, this range is only narrow. We therefore also define a “still acceptable” range of bandgap combinations, which still achieves an efficiency of minimum 80% relative of the maximum at 300 nm. Figure 9 gives guidelines for the sufficient bandgap combinations for an organic triple-junction solar cell.

### 4.3. Subcells with an Unequal Absorption Window 

**Figure 10:**
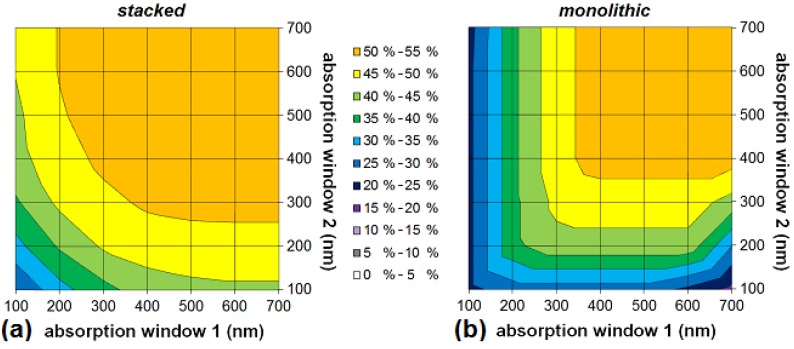
The maximum efficiency in the ideal scenario for an organic stacked (**a**) and monolithic (**b**) tandem cell as a function of the absorption windows of the subcells. “Absorption window 1” refers to the first subcell with the highest absorber bandgap, *i.e.*, the top cell directed at the sun.

We now look at the situation where both subcells of a tandem structure have a different absorption window. In Figure 10 we plot the maximum efficiency for different absorption windows of the subcells. Each data point was calculated using the optimal bandgap configuration for this particular absorption window combination.

One notices that a small absorption window for just one of the subcells of the stacked cell does not lead to a significant decrease in efficiency, as long as the absorption window of the other subcell is wide enough (Figure 10a). If one subcell has an absorption window of only 100 nm or 200 nm, the maximum efficiency is still about 80% or 90% respectively of the absolute maximum obtained in the case of full absorption windows, as long as the other subcell has an absorption window of at least 400 nm.

This does not apply for the monolithic cell (Figure 10b). As soon as one subcell has a low absorption window, the efficiency decreases rapidly. A monolithic cell with an absorption window of 100 nm or 200 nm for the first subcell, and 400 nm for the second subcell, only has a maximum efficiency of less than half and three quarters, respectively, of the absolute maximum for full absorption windows. At an absorption window of 700 nm, the efficiency decreases again because of the limits on the allowed bandgaps (by imposing the absorption window).

Again, the plots show for both configurations that absorption windows of more than 400 nm are not necessary for achieving good power conversion efficiency. Figure 10a and Figure 10b are not symmetrical: for example, a monolithic tandem cell where the first subcell has an absorption window of 100 nm has a maximum attainable efficiency of 23.5%, whereas if it is the second subcell that has an absorption window of 100 nm, the maximum attainable efficiency is 27.7%. The asymmetry is only minor, but is more present for a monolithic cell.

**Figure 11:**
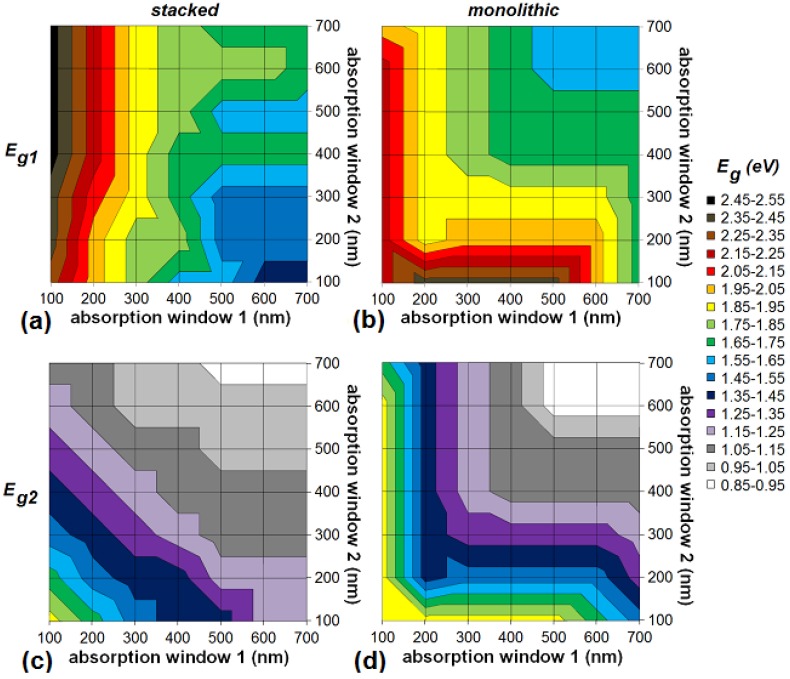
The optimal bandgaps *E_g1_* (**a**,**b**) and *E_g2_* (**c**,**d**) in the ideal scenario for an organic stacked (**a**,**c**) and monolithic (**b**,**d**) tandem cell as a function of the absorption windows of the subcells. “Absorption window 1” refers to the first subcell with the highest absorber bandgap, *i.e.*, the top cell directed at the sun.

Figure 11 shows the optimal bandgap configurations for a stacked and monolithic organic tandem cell with unequal absorption windows. In general, the optimum bandgap of the cells shifts towards higher energies for lower absorption windows. In an organic stacked tandem cell, the optimal bandgap of the first subcell (*E_g1_*) only reaches values higher than 2.0 eV when the absorption window of the first subcell is 200 nm wide or less (Figure 11a). Narrowing the absorption window of the first cell increases the optimal bandgap *E_g1_*. The value of the bandgap *E_g1_* depends mainly on this first absorption window. It is not very dependent on the absorption window of the second subcell. In contrast with the stacked cell, the optimal bandgap *E_g1_* for a monolithic configuration depends on both absorption windows (Figure 11b). The lower the absorption windows of the subcells, the higher the optimal bandgap *E_g1_*.

The optimum of the bandgap of the second subcell (*E_g2_*) is always such that the absorption window of the second subcell borders (or overlaps) the cut-off wavelength of the first subcell (not visible on the figures). This is the case for the stacked as well as the monolithic configuration. Only when the absorption window width of the second subcell is 100 nm or less, there is some significant space between both absorption windows, although never more than 50 nm. Hence, in all optimal bandgap configurations, (nearly) the entire solar spectrum between the outside borders of the absorption windows is absorbed. Please note that in our model, when the absorption windows overlap, the first subcell absorbs all the photons in the overlapping region, the second subcell none.

The optimal bandgap *E_g2_* of the stacked cell (Figure 11c) shifts (quite symmetrically) towards higher energies for lower absorption windows of both the first and the second subcell. The explanation is that the optimal bandgap *E_g2_* is located in such a way that it (almost) borders the absorption window of the first subcell, as mentioned above. This is a satisfying result, taking into account the characteristic narrow absorption window of organics and the difficulty of producing suitable low bandgap organic materials. Unfortunately, this has a negative influence on the efficiency.

**Figure 12:**
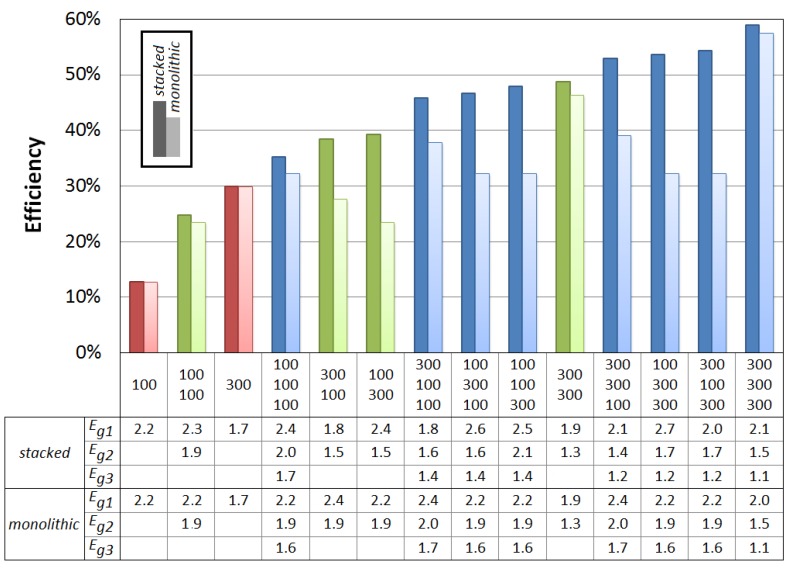
The maximum efficiency in the ideal scenario for an organic stacked and monolithic solar cell as a function of the absorption windows of the subcells. The label “300 100 100” for example indicates a triple-junction whose first subcell has an absorption window of 300 nm and whose second and third subcell have an absorption window of 100 nm. As comparison, we also add the single-junctions (in red) and tandem cells (in green). The optimal bandgaps of the subcells in each configuration are given in eV.

Similar conclusions can be drawn for the triple-junction solar cell with different absorption windows for the three subcells. Again, for both configurations, absorption windows of more than 300 nm are not necessary for achieving good power conversion efficiency of an organic triple-junction solar cell. We therefore take a look at the efficiencies in the ideal scenario for triple-junction cells whose subcells have either a sufficient (300 nm) or a too small (100 nm) absorption window. In Figure 12, the label “300 100 100” for example indicates a triple-junction whose first subcell has an absorption window of 300 nm and whose second and third subcell have an absorption window of 100 nm. As comparison, we also add the single-junctions and tandem cells with those absorption windows to Figure 12.

One notices (Figure 12) that a small absorption window for just one of the subcells of the stacked cell does not lead to a big decrease in efficiency, as long as the absorption windows of the other subcells are wide enough. If one subcell has an absorption window of only 100 nm, the maximum efficiency is still about 85% of the absolute maximum obtained in the case of full absorption windows, as long as the other subcells have an absorption window of at least 300 nm. Even when only one subcell has a sufficient absorption window of 300 nm, and the other 2 have a small absorption window of 100 nm, the efficiency is still 75% relative of the absolute maximum.

Similar to the tandem cell, this does not apply for the monolithic triple-junction cell (Figure 12). As soon as one subcell has a low absorption window, the efficiency decreases rapidly. A monolithic cell with an absorption window of 100 nm for the first subcell, and 300 nm for the other subcells, only has a maximum efficiency of about half of the absolute maximum for full absorption windows.

Figure 12 also shows the optimal bandgaps for the different configurations. Similar conclusions regarding the bandgaps of the triple-junction can be drawn as for the organic tandem cell:

- In general the optimum bandgap of the cells shifts towards higher energies for lower absorption windows.
- The optimum of the bandgap *E_g2_* of the second subcell is always such that the absorption window of the second subcell borders (or overlaps) the cut-off wavelength of the first subcell, and analogous for *E_g3_*. Hence, in all optimal bandgap configurations, (nearly) the entire solar spectrum between the outside borders of the absorption windows is absorbed.

### 4.4. The Maximum Obtainable Efficiency in a More Realistic Situation

For our calculations, we idealized some scalable parameters which were not relevant for determining guidelines for the bandgap combinations and absorption windows for organic tandem and triple-junction solar cells. To estimate the best obtainable efficiency in the near future however, we now assume the following realistic values, which are with the current state of technology nowadays reached in organic photovoltaics [3,24,47]. We assume an *EQE* of 75%, a fill factor *FF* of 75%, and a voltage factor *f* of 70%, with *f* defined by:

(5) $$f=\frac{q\cdot V_{oc}}{E_{i}}$$

We consider that all subcells have the same *EQE*, *FF* and *f*. For the tandem cell, we assume an absorption window of 400 nm. This results in a maximum attainable efficiency of 20.9% and 20.5%, respectively, for stacked and monolithic organic solar cells, both at an optimal configuration (*E_g1_*, *E_g2_*) of (1.7 eV, 1.1 eV). For the triple-junction cell, we assume an absorption window of 300 nm. This results in an efficiency of 23.2% and 22.7%, respectively, for stacked and monolithic cells, at an optimal configuration (*E_g1_*, *E_g2_*, *E_g2_*) of (2.1 eV, 1.5 eV, 1.1 eV) and (2.0 eV, 1.5 eV, 1.1 eV) respectively. Again, several suitable organic materials with those bandgaps are available for the first and second subcell [24,33,49,50]. Because a narrow absorption window is sufficient, the optimal bandgap for the third subcell is now high enough to also have several material options of 1.1 eV bandgap available [24,33,49], e.g., carbazole copolymers [51].

Nevertheless, tandem and triple-junctions of more than 20 % efficiency are still not in range. Why not? Remember that our model is intended to define an acceptable range for the bandgap combinations and absorption windows. We therefore omitted a lot of other factors also influencing the performance of the solar cell, such as carrier mobility and carrier lifetime, active layer thickness, blend composition, morphology of the device, *etc.* [33,52,53]. The organic materials with suitable bandgaps do not have the same properties as the best organic materials like P3HT. Furthermore, not only a good donor, but also the existence of an appropriate good acceptor with ideal energy levels (such as a minimal ΔLUMO with the donor) is important.

## 5. Conclusions and Further Work

We have determined the optimal bandgap configurations of the subcells for an organic tandem and triple-junction solar cell, as well as their acceptable range. The requirements for a close to optimal configuration of the stacked tandem cell are quite broad. This is not the case for the monolithic configuration. An important conclusion is that an absorption window for each subcell of 400 and 300 nm for respectively a tandem and triple-junction cell is more than sufficient. Furthermore, for a stacked organic tandem cell, it is not necessary that both subcells have a large absorption window. This does not apply for the monolithic cell. As soon as one subcell has a low absorption window, the efficiency decreases rapidly.

In our model, we assumed full absorption in each subcell (and consequently leaving the thickness of the subcells aside). We neglected interference and optical coupling of the subcells. As future work, we would like to include the thickness of the layers, and thus the influence of a limited absorption in each subcell, as well as the influence of current matching for the monolithic configurations. Indeed, the active layers in organic solar cells have typically a thickness below 300 nm and, because the sun light has to be considered as coherent on a scale of one to two periods of the incident light wave, the optics of organic solar cells are in most cases dominated by interference effects caused by the reflecting back electrode [21]. Therefore, the position of the subcells to the reflecting electrodes can play an important role in organic multi-junction cells [54].
